# Evaluation of edible polymer coatings enriched with green tea extract on quality of chicken nuggets

**DOI:** 10.14202/vetworld.2016.685-692

**Published:** 2016-07-02

**Authors:** Prathyusha Kristam, Naga Mallika Eswarapragada, Eswara Rao Bandi, Srinivas Rao Tumati

**Affiliations:** 1 Department of Livestock Products Technology, NTR College of Veterinary Science, Gannavaram, Andhra Pradesh, India; 2 Department of Veterinary Public Health & Epidemiology, NTR College of Veterinary Science, Gannavaram, Andhra Pradesh, India

**Keywords:** chicken nuggets, edible coatings, green tea extract, sodium alginate

## Abstract

**Aim:**

The present study was conducted to evaluate the physico-chemical and microbiological characteristics of chicken nuggets coated with sodium alginate (SA) coatings at refrigerated (4±1°C) and frozen (−18±1°C) storage condition at regular periodic intervals.

**Materials and Methods:**

Chicken meat nuggets were separated into three groups: Uncoated control (C), coated with alginate coating (T_1_), and coated with alginate coating incorporated with 1% green tea extract (GTE) (T_2_). The nuggets were analyzed at regular intervals of 5days for refrigerated storage and 15 days for frozen storage period in terms of pH, 2-thiobarbituric acid value (TBA), peroxide value (PV), total plate count (TPC), water loss, and sensory characteristics.

**Results:**

The results indicated that the nuggets coated with alginate-based coatings effectively reduced the spoilage as indicated by pH, TBA, and PVs. pH values of the formulations ranged from 6.15 to 6.34 at refrigerated storage temperature (4±1°C) and 6.49-6.71 at frozen storage temperature (−18±1°C). TBA value of the treatments ranged from 1.28 to 1.54 mg MDA/kg and 1.34 to 1.50 mg MDA/kg under refrigerated and frozen storage temperatures, respectively. Color, flavor, juiciness, tenderness, and overall acceptability of the nuggets differed significantly (p<0.05) with the coated nuggets. The coated nuggets were well acceptable upto 15 days at refrigerated storage temperature (4±1°C) and upto 75 days at frozen storage temperature (−18±1°C). Nuggets coated with GTE incorporated coating solution had a lower TBA-reactive substances values, PVs, and TPCs when compared to the nuggets coated with SA and the control group.

**Conclusion:**

Study revealed that incorporation of edible coatings with antioxidants, namely, GTE at 1% level had a significant effect in reducing the fat oxidation. The samples recorded a shelf life of 15 days under refrigerated storage when compared to their controls with 10 days of storage period and 75 days under frozen storage against controls with 60 days. T_1_, T_2_, and T_3_ formulations had higher sensory scores in comparison to the controls. Overall acceptability scores of T_1_ were higher when compared to the other formulations.

## Introduction

The perishable nature of meat limits its sensory quality and influences the shelf life. Packaging of the meat is one of the methods adopted to retard the spoilage and deliver the foods safely to the consumers. Synthetic packaging films have led to serious ecological problems due to their non-biodegradability. Hence, there is a need to search for alternative packaging technologies with advantages over synthetic packaging materials.

At present, there is a renewed interest in the development of edible films which can be attributed to environmental concerns over disposal of non-renewable food packaging materials and the difficulties in disposinghuge waste volumes generated by non-biodegradable food packaging. These issues have motivated the study of biopolymers as material to be used as edible coatings. There is a lot of work going on in this era on edible coatings of vegetables and fruits [1-3], which are slowly being extended to the application on meats [4] and also at different atmosphere conditions [5].

Edible coatings prepared from polysaccharides, proteins, and lipids may serve as oxygen and/or moisture barriers and can be used to maintain food quality. Collagen casings were the earliest used protein packaging materials for meat [6]. Natural extracts, which possess antioxidant properties, were being added to enrich the films or coatings [7] with green tea extract (GTE) made to extend the shelf life of fish [8] and grape seed extract [9].

Film forming properties of alginate are related to its ability to form strong gels or insoluble polymers in the presence of polyvalent metal cations such as calcium (Ca2+) [10]. Alginate coatings, as other polysaccharide-based coatings, present low oxygen permeabilities due to their ordered hydrogen-bonded network structure, and it has been widely used in coating of meat products due to its scarifying agent and protects against lipid oxidation [11] and for the addition of ingredients to extend the quality of the coating [12].

Green tea (*Camellia sinensis*) is one of the mostpopular and known natural antioxidants. It contains several polyphenolic components with antioxidant and pro-oxidant properties, particularly flavonoids, but the predominant active components are the flavanol monomers known as catechins, where epigallocatechin-3-gallate and epicatechin-3-gallate are the most effective compounds [13] and decreases the levels of oxidized proteins and lipids [14]. It contains a wide variety of other components such as flavones, phenolic acids and depsides, carbohydrates, alkaloids, minerals, vitamins, and enzymes [15]. GTE upon direct addition into the food may alter the palatability characteristics of the product as it contains different volatile flavor components [16]. Alternatively, it can be effectively incorporated into coatings which can be used as food wraps.

The meat industry is increasingly searching for natural solutions to minimize oxidative rancidity and extend the shelf life of meat products rather than synthetic additives. Thus, the research for alternative methods to retard oxidative processes in meat has led to research on alternative natural antioxidants.

The aim of this study was to develop an edible alginate-based coating incorporated with GTE (*C. sinensis*) (70% catechins) and evaluate physico-chemical and microbiological characteristics of coated chicken nuggets at refrigeration (4±1°C) and frozen (−18±1°C) storage condition.

## Materials and Methods

### Ethical approval

Permission was obtained from University Ethics Committee, SVVU, Tirupathi.

### Materials

Fresh boneless broiler chicken meat was procured from local market. All chemicals utilized for evaluation of the quality characteristics of chicken meat nuggets (glycerol is food grade) were procured from Himedia Laboratories Pvt. Ltd., India. Natural antioxidants such as lyophilized green tea powder of food grade (70% catechins) were purchased from Kamco Pharmaceuticals, Hyderabad, Andhra Pradesh, India.

### Methodology

#### Preparation of nuggets

Chicken meat nuggets were prepared by adding minced meat and other ingredients of recipe in a sequential order. Minced meat was chopped in bowl chopper (Scharfen) by adding the ingredients, namely, salt, fat, binder (corn flour), spice and chili powder, condiments, and chilled water in the form of crushed ice at 1.8%, 5.0%, 3.0%, 2.2%, 4.0%, and 10.0%, respectively, and during chopping, the emulsion was maintained at 10-12°C by addition of crushed ice. The emulsion was filled in the steel mold and cooked at 75±2°C for 45 min, then made into pieces to form nuggets.

#### Preparation and application of coating solutions

Coating solutions were prepared with sodium alginate at a concentration of 2% (w/v) in distilled water. Sodium alginate (SA) was melted with continuous stirring to allow hydrolyzation by heating to 90°C on a magnetic stirrer. Glycerin was used as plasticizer at 4 % level, and it was added after cooling the hydrolyzed solution to 70°C and GTE at 1% level was also added slowly with continuous stirring to the above solution. Two types of edible coatings were produced, i.e., without additives (T_1_) and with incorporation of 1% GTE (T_2_). Chicken nuggets prepared according to the method described were coated with the coating solutions. Chicken nuggets were dipped in the coating solutions for 1 min; then, they were drained of excess solution for 30 s followed by dipping in 2% aqueous calcium chloride solution for 30s. Coated nuggets were kept in hot air oven at 40°C for 30min for the efficient casting of coating over the nuggets.

The coated nuggets with SA alone (T_1_), SA, and GTE (T_2_) were packaged along with uncoated nuggets as control (C) in low-density polyethylene covers. They were labeled and stored at refrigeration temperature (4±1°C) and frozen temperature (−18±1°C). The products were analyzed at regular intervals of 5 and 15 days at refrigeration and frozen temperatures, respectively, for physico-chemical, sensory, and microbiological qualities.

### Physico-chemical characteristics

#### Proximate composition

The percent moisture, fat, and crude protein were estimated [17].

#### pH

pH of the preparation was estimated by following the method of Trout *et al*. [18] using a digital pH meter of (Oakton Instruments, USA).

#### 2-thiobarbituric acid reactive substances (TBARS)

The distillation method outlined by Lawlor *et al*. [19] was followed for the determination of TBARS values.

#### Peroxide value (PV)

PV of the product was determined by standard technique [17].

### Total plate count (TPC)

For microbiological analysis, about 5 g of representative sample was homogenized with 45 ml of 0.1% sterile peptone water and serial dilutions were made using 0.1% sterile peptone water. The TPC were enumerated on duplicate pour plates of plate count agar which were incubated a 37°C for 48 h. Counts were expressed as log CFU/g of sample [20].

### Water loss analysis

The water loss was estimated as described by Lu *et al*. [21]. The percent weight loss relative to the initial weight was calculated by weighing the samples every 5 days in duplicate.

### Sensory evaluation

The chicken meat nuggets were cooked and subjected to a six-member taste panel for sensory evaluation to evaluate color, appearance, flavor, juiciness, tenderness, and overall acceptability on a 9 point hedonic scale.

### Statistical analysis

The data were subjected the statistical analysis using SPSS MAC, version 22.0, SPSS Chicago (USA). The entire experiment was repeated six times to reduce the standard error.

## Results and Discussion

### pH

The pH values of the treatments during storage period were presented in Tables-1 and 2. There was a significant difference (p<0.05) between the pH values of coated and control chicken meat nuggets. The lower pH values of the coated nuggets might be due to the effect of SA coating. During storage, irrespective of the formulations, the pH values increased which may be due to the microbial production of alkalinizing substances; however, the rate of increase in coated nuggets was at a slower pace, which can be attributed to the activity of the ingredients in the coatings. The results indicated that the GTE when incorporated in alginate-based coatings was effective in decreasing the rate of increase in pH indicating extension of shelf life of the coated nuggets. This observation was similar to in beef patties [22] and on shelf life extension of refrigerated bream with SA coatings [23].

**Table-1:** pH, TBA, and PV (mean±SE) values of chicken meat nuggets as influenced by different coatings during refrigerated (4±1°C) storage.

Days	pH			TBA (mg MLD/Kg)			PV mEq/kg fat		
		
	C	T_1_ (SA)	T_2_ (SA+GTE)	C	T_1_ (SA)	T_2_ (SA+GTE)	C	T_1_ (SA)	T_2_ (SA+GTE)
0	6.25±0.05	5.96±0.04	5.93±0.03	0.98±0.04	1.00±0.02	0.98±0.01	0.68±0.03	0.69±0.04	0.68±0.01
5	6.33±0.02	6.15±0.03	6.12±0.04	1.21±0.03	1.11±0.05	1.07±0.03	1.42±0.02	1.12±0.04	0.9±0.03
10	6.38±0.03*	6.24±0.04	6.26±0.02	1.86±0.03*	1.47±0.04	1.41±0.02	2.52±0.04*	1.88±0.03	1.42±0.05
15			6.29±0.02			1.64±0.04			2.42±0.02

* Spoiled, p<0.05.

Means bearing no superscript do not differ significantly. SA=Sodium alginate, GTE=Green tea extract, GSE=Grape seed extract, TBA=Thiobarbituric acid, PV=Peroxide value, SE=Standard error

**Table-2:** pH, TBA, PV (mean±SE) values of chicken meat nuggets as influenced by different coatings during frozen (18±1°C) storage.

Days	pH			TBA (mg MLD/kg)			PV mEq/kg fat		
		
	C	T_1_ (SA)	T_2_ (SA+GTE)	C	T_1_ (SA)	T_2_ (SA+GTE)	C	T_1_ (SA)	T_2_ (SA+GTE)
0	6.25±0.05	5.96±0.04	5.93±0.03	0.98±0.04	1.00±0.02	0.98±0.01	0.68±0.03	0.69±0.04	0.68±0.01
15	6.32±0.01	6.29±0.03	6.27±0.02	1.22±0.02	1.14±0.01	1.11±0.03	0.89±0.02	0.77±0.01	0.76±0.03
30	6.44±0.02	6.41±0.03	6.38±0.04	1.34±0.04	1.29±0.05	1.20±0.03	1.33±0.04	1.25±0.03	1.15±0.05
45	6.56±0.02	6.49±0.02	6.44±0.03	1.45±0.03	1.39±0.04	1.34±0.04	1.52±0.05	1.47±0.04	1.38±0.03
60	6.72±0.03*	6.53±0.04	6.51±0.02	1.74±0.02*	1.59±0.02	1.43±0.03	2.68±0.02*	2.32±0.03	2.24±0.02
75		6.81±0.04*	6.57±0.03		1.66±0.04*	1.61±0.05		2.70±0.04*	2.59±0.02
90			6.70±0.01*			1.76±0.02*			2.87±0.02*

* Spoiled, p<0.05,

Means bearing no superscript do not differ significantly. SA=Sodium alginate, GTE=Green tea extract, GSE=Grape seed extract, TBA=Thiobarbituric acid, PV=Peroxide value, SE=Standard error

### TBA value

TBARS values had been widely used to estimate the extent of lipid oxidation and presence of TBARS due to second stage auto-oxidation during which peroxides are oxidized into aldehyde and ketone. The TBA values were represented in Tables-1 and 2.

Coated nuggets recorded significantly (p<0.05) lower values when compared to the control group. This might be due to the SA coating having a base of strongly cross-linked polymers. The values of T_2_ were significantly (p<0.05) lower than that of uncoated samples and as well as T_1_ throughout the storage period indicating the efficacy of antioxidants GTE added into the coating in its efficacy in inhibiting lipid oxidation. GTEs contain polyphenols that have been reported to act as free radical scavengers to terminate the radical chain reactions that occur during the oxidation of triglycerides in food system [24]. The alginate-based film layers on the surface of the product might have resisted oxygen diffusion thus may have retarded lipid oxidation. The TBA values of treated and control nuggets increased continuously during storage irrespective of the temperature of storage which may be attributed to partial dehydration of the product and increased oxidation of unsaturated fatty acids. Similar pattern in results was observed for pre-cooked ground-beef patties packaged in edible starch-alginate-based films [22,25], in alginate coated beef patties and with chitosan coatings on herring cod [26], with whey protein coating on sausages [27], and with SA-based coatings incorporated with tea polyphenols and vitamin C on the qualities and shelf life of refrigerated bream [23].

### PV

PV is a measure of the concentration of peroxides and hydroperoxides formed during auto-oxidation of unsaturated fats. Peroxides are the intermediates in auto-oxidation reaction which is free radical reaction involving oxygen which leads to deterioration of fats and oils producing off flavors and off odors. PV thus is useful for assessing the extent to which spoilage has advanced. PVs were presented in Tables-1 and 2. There was a significant difference (p<0.05) between coated and uncoated chicken meat nuggets. However, in the T_2_ formulation which was being added with strong antioxidants, GTE was significantly (p<0.05) lower in their PV when compared to control and T_1_ formulations. Peroxides begin to disintegrate leading to production of aldehydes, ketones, and TBA; but in coated samples due to the properties of ingredients in edible films, the increase of TBA and peroxides was prevented. Irrespective of the temperature of storage and treatments the PV increased with increasing storage period. However, the antioxidant-enriched coated nuggets had a lower rate of increase in PV than the controls. These results were in agreement while using chitosan coatings on herring and Atlantic cod [28], whey protein coating on sausages [27], on soyprotein isolated coatings on beef [29], and on whey protein coated gutted kilka during frozen storage [30].

### TPC

The TPC of treatment samples was summarized in Tables-3 and 4. TPC values were significantly (p<0.05) lower in coated samples in comparison to the control samples. This might be due to the ability of SA to produce gels due to its glucuronic acid and mannuronic acids. This property resulted in the formation of semipermeable layer on the product that can reduce microorganism infiltration into coated samples. During storage, the TPCs increased with increasing storage period. However, lower counts of treatment samples were noticed in comparison with controls. Further, TPC of T_2_ was significantly (p<0.05) lower than the control and T_1_ formulations. Unacceptable TPC values were found in control on day 10 under refrigerated storage temperature and on day 60 under frozen storage temperature. Statistically significant (p<0.05) difference in mean TPC values between control and treatment samples was observed at day 10 at refrigerated storage temperature and day 60 under frozen storage temperature. However, there was an increase in the shelf life of 5 days at refrigerated storage temperature and 15 days at frozen storage temperatures although trends indicated marginal low microbial load as a result of addition of GTE in alginate coatings. This low microbial load might be due to the effect of polyphenols that are present in tea which was proved to possess certain antimicrobial activity *in vitro* although no specific studies on meat products have been made. The results indicated that plant extract-based antioxidants, namely, GTE may exhibit cooperative inhibitory activity on bacteria, but at the tested levels, they were not having the potential inhibitory activity. Thus, it can be stated that the usage of plant-based antioxidants and antimicrobials can be alternatives for chemicals used in food preservation. The results were similar with incorporation of nisin and chelators into protein and polysaccharide-based films inhibiting growth of *Salmonella* on poultry skin [31], with whey protein isolate containing sorbic acid and p-aminobenzoic acid in inhibiting the growth of *Listeria monocytogenes*, *Escherichia coli* O157H7, and *Salmonella* Typhimurium DT104 on a non-selective plating medium [32] andincorporation ofGTE in active film from chitosan to extend the shelf life of pork sausages and concluded that samples wrapped with film showed lower total count, yeasts, and molds and lactic acid bacteria (log CFU/g) when compared to control and those wrapped with chitosan film alone during storage at 4°C [33].

**Table-3:** TPC and water loss analysis (mean±SE) values of chicken meat nuggets as influenced by different coatings during refrigerated (4±1°C) storage.

Days	TPC			Water loss analysis		
	
	C	T_1_ (SA)	T_2_ (SA+GTE)	C	T_1_ (SA)	T_2_ (SA+GTE)
0	4.54±0.04	4.53±0.03	4.01±0.05	0.00±0.0	0.00±0.0	0.00±0.0
5	4.84±0.04	4.52±0.03	4.11±0.02	0.14±0.02	0.11±0.03	0.13±0.04
10	5.83±0.01*	4.95±0.02	4.49±0.04	0.23±0.05	0.18±0.04	0.18±0.03
15		5.03±0.04*	4.77±0.03	0.29±0.02	0.24±0.03	0.25±0.03

* Spoiled, p<0.05,

Means bearing no superscript do not differ significantly. SA=Sodium alginate, GTE=Green tea extract, GSE=Grape seed extract, TPC=Total plate count, SE=Standard error

**Table-4:** TPC and water loss analysis (mean±SE) values of chicken meat nuggets as influenced by different coatings during frozen (−18±1°C) storage.

Days	TPC			Water loss analysis		
	
	C	T_1_ (SA)	T_2_ (SA+GTE)	C	T_1_ (SA)	T_2_ (SA+GTE)
0	4.54±0.04	4.53±0.03	4.01±0.05	0.00±0.0	0.00±0.0	0.00±0.0
15	4.62±0.02	4.53±0.03	4.23±0.04	0.14±0.03	0.11±0.02	0.12±0.01
30	4.85±0.03	4.62±0.03	4.33±0.04	0.18±0.03	0.15±0.04	0.14±0.05
45	4.98±0.03	4.72±0.04	4.52±0.04	0.26±0.03	0.18±0.03	0.19±0.02
60	5.73±0.03*	4.95±0.04	4.71±0.04	0.33±0.04	0.22±0.05	0.21±0.03
75		5.08±0.03*	4.92±0.02	0.40±0.01	0.24±0.02	0.25±0.05
90			5.07±0.04*	0.48±0.02	0.28±0.03	0.27±0.01

* Spoiled. p<0.05,

Means bearing no superscript do not differ significantly. SA=Sodium alginate, GTE=Green tea extract, GSE=Grape seed extract, TPC=Total plate count, SE=Standard error

### Water loss analysis

Water loss of the treatments along with control was depicted in Tables-3 and 4. There was significant (p<0.05) difference in water loss between coated and uncoated chicken meat nuggets. However, no significant (p>0.05) difference between the treatment groups was observed. The lower water loss for the coated nuggets might be due to the SA coating which forms an insoluble polymer through cross-linking that can control the loss of water and reduce dehydration. Further, the gel coating could act as a sacrificing agent to reduce the desiccation of the product [23]. Chelating of calcium ions and decreasing the link of protein by producing ion bridge increased the single and multiple layer water storage in myofibrils thus preventing dehydration of tissue in SA-coated samples. Irrespective of treatment and temperature of storage water loss significantly (p<0.05) increased during the storage period. This increase might be due to loss of water in the form of water vapor. However, the rate of loss of moisture in coated nuggets was lower when compared to the uncoated controls. This canbe attributed to the development of desiccated surface layer overcoating in cold storage which produces a resistance to mass transfer thus bringing about a less water loss. Similar results were observed with chitosan coatings to reduce water loss from herring and Atlantic cod [28] and on refrigerated bream with alginate-based coatings [23].

### Sensory evaluation

**Figure-1:**
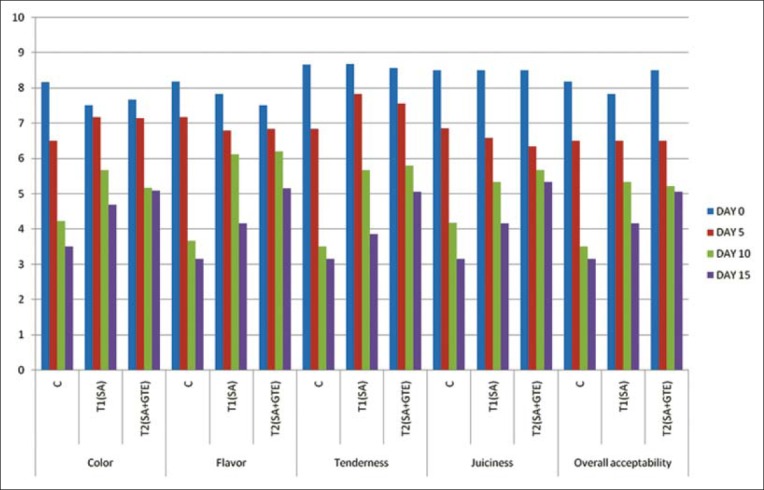
Sensory evaluation of chicken nuggets at refrigerated storage.

**Figure-2:**
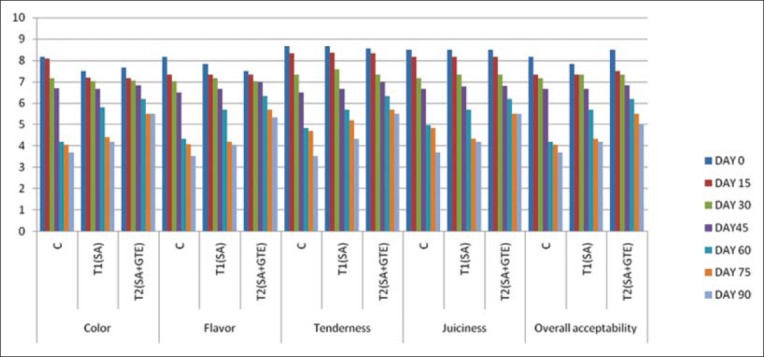
Sensory evaluation of chicken nuggets at frozen storage.

The sensory evaluation results were summarized in Figures-1 and 2. The appearance of the product to the consumer is greatly determined by the color of the product. When the product was applied with edible coatings, the color of the coating and its effect on the product upon cooling, depends on the ingredients in the coating and final color of the cooked product and pigmentary changes that take place during cooking. The formulation of chicken meat nuggets coated with alginate-based coating recorded significantly (p<0.05) higher mean color scores than the control nuggets. The higher scores would have been due to the properties of polysaccharide films, i.e., preventing dehydration, oxidative rancidity, and surface browning. Irrespective of the type of coating and storage temperature the color scores significantly (p<0.05) decreased with increase in the storage period. The changes in color qualities might be due to microbial growth and lipid oxidation during storage.

Flavor is a complex sensation involving odor and taste. It is important both esthetically and physiologically for improving the secretion of digestive juices. Many types of heat induced reactions of cooking lead to the production of meat flavors. Sensory evaluation results showed that flavor scores of coated nuggets were significantly (p<0.05) higher than the control nuggets. The better flavor scores of coated nuggets may be attributed to the coating of SA which was considered as flavoring agent [34]. When compared to T_1_, the lower scores of T_2_ even though were non-significant can be attributed to the retarded oxidation due to the antioxidants. Irrespective of the type of coating and storage temperature the flavor scores significantly (p<0.05) decreased with increasing storage period. This may be due to the growth of microorganisms and lipid oxidation during storage.

Tenderness in the meat products is rated as most important by the average consumer and appears to be sought at the expense of flavor and color. The tenderness of the coated product was significantly (p<0.05) higher than their uncoated counterparts. Similar to color and flavor irrespective of the type of coating and storage temperature, the tenderness scores significantly (p<0.05) decreased with increasing storage period. This might be due to the relative reduction in moisture content and thus reduction in juiciness of the product.

The juiciness in meat has two organoleptic components; the first is the impressionof wetness in first chews due to the rapid release of fluid and second is the sustainability of juiciness due to the stimulatory effect of fat on salivation. The overall mean juiciness scores of nuggets coated with SA base were significantly (p<0.05) lower than uncoated nuggets. The higher juiciness scores might be due to retention of more moisture in the coated product. In addition to this, prevention of tissue dehydration by myofibril denaturation and hydrophilic nature of SA molecules may also aid in increased juiciness of the nuggets and irrespective of the type of coating and storage temperature the juiciness scores significantly (p<0.05) decreased with increasing storage period. This might be due to the relative reduction in moisture content and thus reduction in juiciness of the product.

The mean overall acceptability values of coated chicken meat nuggets were significantly (p<0.05) higher than that of the control formulation. Superior scoring in respective of color, flavor, tenderness, and juiciness had reflected in higher overall acceptability scores for the coated formulation. However, the overall acceptability scores of T_2_ formulation were slightly higher than T_1_ formulation. This can be attributed to the reduced loss of volatile compounds while cooking in these products. Irrespective of the type of coating and storage temperature the overall acceptability scores significantly (p<0.05) decreased with increasing storage period. This might be due to the lower scores of color, flavor, tenderness, and juiciness.

Sensory evaluation scores were in agreement with sodium alginate-based coatings on refrigerated bream [23], with chitosan green tea film [33], and with sodium alginate coating at 2% level significantly (p<0.05) improved the overall appearance and color, juiciness, flavor, texture, and overall palatability of beef patties [22].

### Proximate composition

The results of proximate composition of chicken meat nuggets were represented in Table-5. The percent moisture content of the coated nuggets was higher than the uncoated ones. This might be due to the retention of moisture content in the nuggets. This canbe attributed to the fact that gel coating which acts as sacrificing agent, i.e., moisture in the gel evaporates before any significant desiccation of the enrobed food [35].

**Table-5:** Proximate composition (mean±SE) of chicken meat nuggets as influenced by different coatings.

Parameter	C	T_1_ (SA)	T_2_ (SA+GTE)
Moisture	68.75±0.05^a^	70.54±0.04^b^	72.10±0.05^b^
Protein	19.92±0.07^x^	19.88±0.01^x^	19.87±0.06^x^
Fat	3.35±0.14^a^	3.38±0.13^a^	3.37±0.12^a^

*Spoiled.p<0.05. Means bearing no superscript do not differ significantly. SA=Sodium alginate, GTE=Green tea extract, GSE=Grape seed extract, SE=Standard error

There was no difference in the protein and fat content of the coated and uncoated product indicating that the coatings did not affect the protein and fat content of the product. Similar results were also observed with coating of beef patties [22].

## Conclusion

The salient features of the study revealed that incorporation of edible coatings with GTE at 1% level had significant effect in reducing the fat oxidation. The samples recorded a shelf life of 15 days under refrigerated storage when compared to their controls with 10 days of storage period and 75 days under frozen storage against controls with 60 days. Overall acceptability scores of T_2_ were higher when compared to the other formulations. The higher sensory, microbiological, and biochemical qualitiesof the coated formulations revealed that coating of meat products with edible coatings can effectively inhibit lipid oxidation and microbial growth, thus extending the shelf life of the product and suggested potential application of edible film/coatings as antioxidant carriers and biodegradable packaging material, to extend the shelf life of the coated product.

## Authors’ Contributions

PK: Preparation of chicken nuggets and coating with antioxidants, analysis of physico-chemical and microbiological characteristics of chicken nuggets, recording the values and preparation of manuscript. NME: Design of the experiment, technical help in preparation of antioxidant solution and in coating them on nuggets and correction of manuscript. ERB: Correction of manuscript and data analysis. SRT: Correction of manuscript. All the authors read and approved the final manuscript.
